# Inhibition of NLRP3 Inflammasome Pathway by Butyrate Improves Corneal Wound Healing in Corneal Alkali Burn

**DOI:** 10.3390/ijms18030562

**Published:** 2017-03-05

**Authors:** Fang Bian, Yangyan Xiao, Mahira Zaheer, Eugene A. Volpe, Stephen C. Pflugfelder, De-Quan Li, Cintia S. de Paiva

**Affiliations:** 1 Ocular Surface Center, Department of Ophthalmology, Baylor College of Medicine, Houston, TX 77030, USA; bftongji@hotmail.com (F.B.); yangyanx@bcm.edu (Y.X.); mzaheer@bcm.edu (M.Z.); eugenevolpe@yahoo.com (E.A.V.); stevenp@bcm.edu (S.C.P.); dequanl@bcm.edu (D.-Q.L.); 2 Department of Ophthalmology, the Second Xiangya Hospital, Central South University, Changsha 410011, Hunan Province, China

**Keywords:** alkali injury, NLRP3 inflammasome, sodium butyrate, β-hydroxybutyric acid

## Abstract

Epithelial cells are involved in the regulation of innate and adaptive immunity in response to different stresses. The purpose of this study was to investigate if alkali-injured corneal epithelia activate innate immunity through the nucleotide-binding oligomerization domain-containing protein (NOD)-like receptor family pyrin domain containing 3 (NLRP3) inflammasome pathway. A unilateral alkali burn (AB) was created in the central cornea of C57BL/6 mice. Mice received either no topical treatment or topical treatment with sodium butyrate (NaB), β-hydroxybutyric acid (HBA), dexamethasone (Dex), or vehicle (balanced salt solution, BSS) quater in die (QID) for two or five days (d). We evaluated the expression of inflammasome components including NLRP3, apoptosis-associated speck-like protein (ASC), and caspase-1, as well as the downstream cytokine interleukin (IL)-1β. We found elevation of NLRP3 and IL-1β messenger RNA (mRNA) transcripts, as well as levels of inflammasome component proteins in the alkali-injured corneas compared to naïve corneas. Treatment with NLRP3 inhibitors using NaB and HBA preserved corneal clarity and decreased NLRP3, caspase-1, and IL-1β mRNA transcripts, as well as NLRP3 protein expression on post-injury compared to BSS-treated corneas. These findings identified a novel innate immune signaling pathway activated by AB. Blocking the NLRP3 pathway in AB mouse model decreases inflammation, resulting in greater corneal clarity. These results provide a mechanistic basis for optimizing therapeutic intervention in alkali injured eyes.

## 1. Introduction

Chemical injuries to the eye represent between 11.5% and 22.1% of ocular injuries [1], often resulting in an aggressive inflammatory response that can impair corneal re-epithelization, promote keratolysis, and lead to globe perforation at the acute stage. Despite medical and surgical management, most ocular chemical injuries still result in loss of vision or the eye. It is possible that the poor therapeutic response stems largely from an incomplete understanding of the host inflammatory response.

Although the mechanisms initiated in wound healing have not been fully elucidated, there is increasingly evidence that epithelial cells serve not only as an effective barrier against most microorganisms, but also are central participants in the bridging of innate and adaptive immunity [2,3]. The innate immune response relies on evolutionarily ancient germline-encoded receptors, the pattern-recognition receptors (PRRs). Pattern-recognition receptors are expressed by a variety of cells to identify microbial pathogens known as pathogen-associated molecular patterns (PAMPs) and to recognize cell components that are released during cell damage or death known as damage-associated molecular patterns (DAMPs). The main families of PPRs, Toll like receptors (TLRs) and nucleotide-binding oligomerization domain-containing protein (NOD)-like receptors (NLRs), have been shown to be expressed on certain epithelial cells [4]. Airway epithelial cells express a variety TLRs that help them to sense bacterial, fungal, or viral exposure and respond accordingly by secreting a large array of molecules that initiate adaptive immune response [5]. Human corneal epithelial cells also express a variety of TLRs (TLR1, TLR2, TLR3, TLR4, TLR5, TLR6, and TLR9) that may help them mount an adequate response to microbial exposure and other environment stimuli such as oxidative and desiccating stress [6,7,8].

NOD-like receptor family pyrin domain containing 3 (NLRP3), a major inflammatory innate defense mechanism, participates in inflammasome formation through the recruitment of the adapter apoptosis-associated speck-like protein (ASC) with subsequent activation of caspase-1, which leads to secretion of interleukin (IL)-1β or IL-18. NOD-like receptor family pyrin domain containing 3 inflammasome (NLRP3) has been described as an innate sensor of host-derived DAMPs that are released following tissue injury or cell death to activate pro-inflammatory pathways [9,10]. The role of the innate immune response of corneal epithelial cells to sterile wounds is still unknown.

Sodium butyrate (NaB) is a fatty acid that is produced by fermentation of dietary fiber by anaerobic bacteria in the colon [11]. Sodium butyrate has been shown to block inflammasome-mediated inflammatory disease in experimental models of obesity-induced inflammation [12]. Ketone body β-hydroxybutyric acid (HBA) has a similar chemical structure as NaB and was also reported to inhibit NLRP3 activation [12], and has been shown to decrease apoptosis of rat corneal epithelia in dry eye conditions [13]. Therefore, we hypothesize that alkali burns may activate the inflammasome and that inhibition of this pathway with NaB and HBA would improve the fate of injured corneas by suppressing inflammation.

Here, we show that a novel innate immune signaling pathway (NLRP3–ASC–caspase-1–IL-1β) is activated in corneal epithelial cells by alkali burn. Blocking the NLRP3 pathway reduced inflammation, leading to improved wound healing and corneal clarity.

## 2. Results

### 2.1. Corneal Alkali Burn Upregulates NLRP3 and Adaptor Protein ASC in Corneal Epithelium

**Figure 1:**
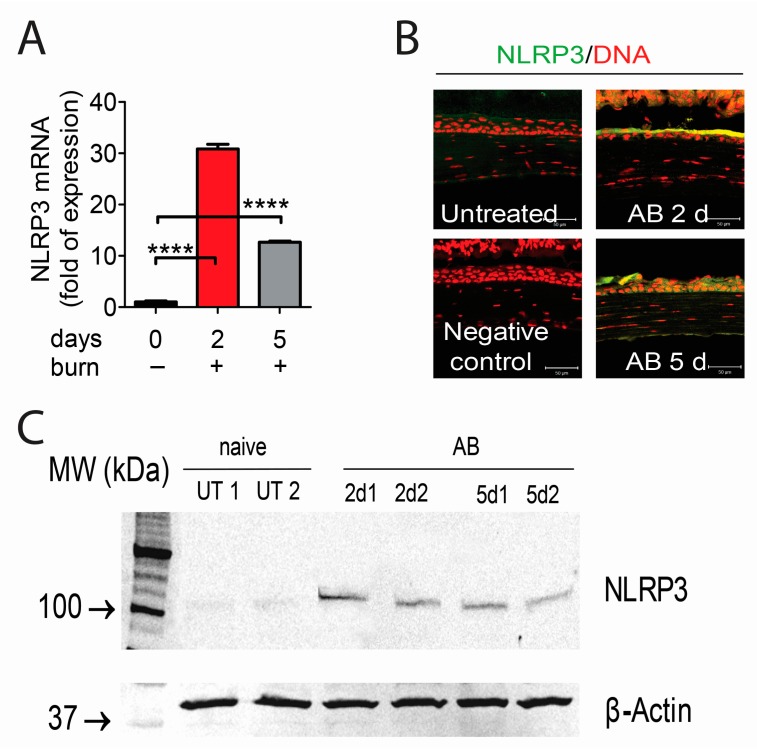
Upregulation of nucleotide-binding oligomerization domain-containing protein (NOD)-like receptor family pyrin domain containing 3 (NLRP3) expression by corneal epithelium in animals subjected to alkali burn (AB) at 2 days (2 d) and 5 days (5 d) post-injury. (**A**) Gene expression analysis of NLRP3 messenger mRNA (mRNA) transcript in whole cornea of animals subjected to alkali burn (*n* = 8 animals/group). Graphs show means ± standard error of the mean (SEM). **** *p* < 0.0001; (**B**) Representative merged digital images of laser scanning confocal microscopy of corneas cryosections immunostained for NLRP3 (green) with propidium iodide nuclei counterstaining (DNA in red) in corneas subjected to alkali burn (images are representative of *n* = 6 animals/group). Scale bar: 50 µm; (**C**) Representative digital images of western blot of NLRP3 and β-actin in cornea epithelium of animals subjected to alkali burn for 2 and 5 days. Each lane is a different sample (*n* = 12 animals/3 samples/group). MW: Molecular weight; UT: untreated control; 2 d and 5 d refer to mice subjected to alkali burn and euthanized 2 or 5 days post-injury, respectively (1 and 2 indicate different samples).

To determine the NLRP3 inflammasome expression in the alkali burned cornea, we first assessed the expression of NLRP3 mRNA transcript by real-time PCR using whole corneas harvested from 2 or 5 days post-alkali burns and naïve corneas as a comparator. As shown in Figure 1A, there was a remarkable increase in NLRP3 transcripts at two days (up to a 30-fold) and five days (up to a 10-fold) in wounded corneas compared to normal corneas.

Next, immunoreactivity of corneas to NLRP3 was evaluated by immunostaining (Figure 1B). Minimal levels of NLRP3 were present in the control corneas, while increased immunoreactivity against NLRP3 was observed in the corneal epithelium of wounded corneas at both 2 and 5 days post-alkali burns. Based on the immunostaining results which showed NLRP3 expression exclusively in the corneal epithelial layer, we repeated the experiment, but only collected corneal epithelium for western blot. Consistent with PCR and immunostaining results, the protein production levels of NLRP3 were increased at 2 days post-injury and slightly decreased at 5 days, but remained elevated compared to the naïve corneas (Figure 1C).

**Figure 2:**
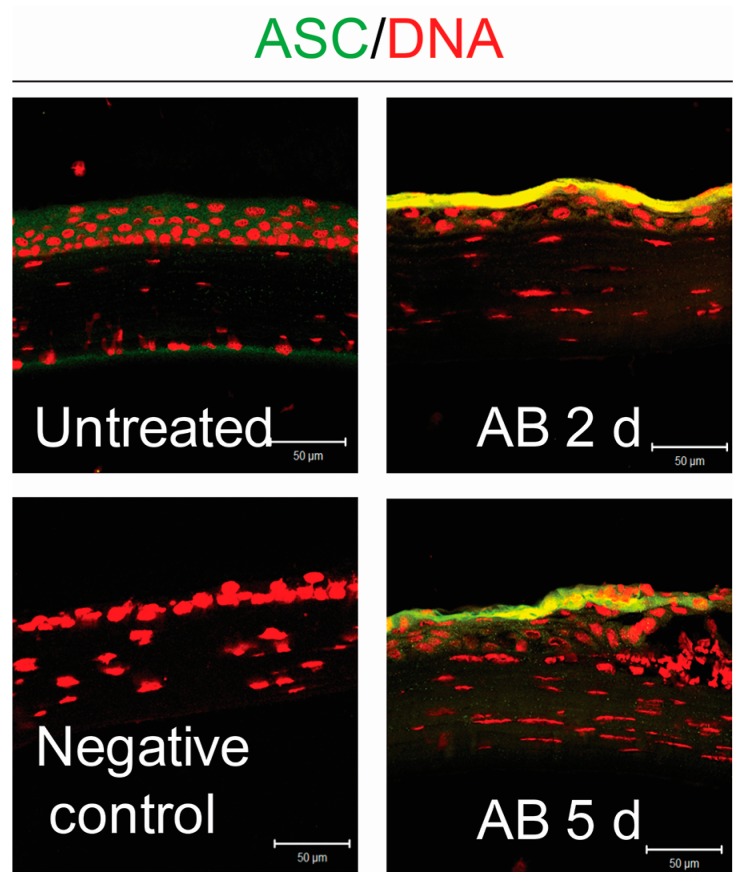
Upregulation of apoptosis-associated speck-like protein (ASC) in corneal epithelium of animals subjected to alkali burns (AB) at 2 days (2 d) and 5 days (5 d) post-injury. Representative merged digital images of laser scanning confocal microscopy in corneal cryosections immunostained for ASC (green) with propidium iodide nuclei counterstaining (DNA in red) in corneas subjected to alkali burn (*n* = 6 animals/group). Scale bar: 50 µm.

Upon activation, NLRP3 associates with the adaptor protein ASC to recruit pro-caspase-1. In order to investigate whether the adapter protein ASC increased in the wounded corneas, we evaluated ASC expression by immunostaining in frozen sections. In contrast to normal control, ASC expression increased both at 2 and 5 days post-injury (Figure 2).

These data demonstrate that both NLRP3 and its adaptor protein ASC in cornea epithelial cells are upregulated following alkali injury, and prompted us to investigate the role of NLRP3 inflammasome in corneal alkali injuries.

### 2.2. Caspase-1 Activation in Alkali-Injured Corneas

**Figure 3:**
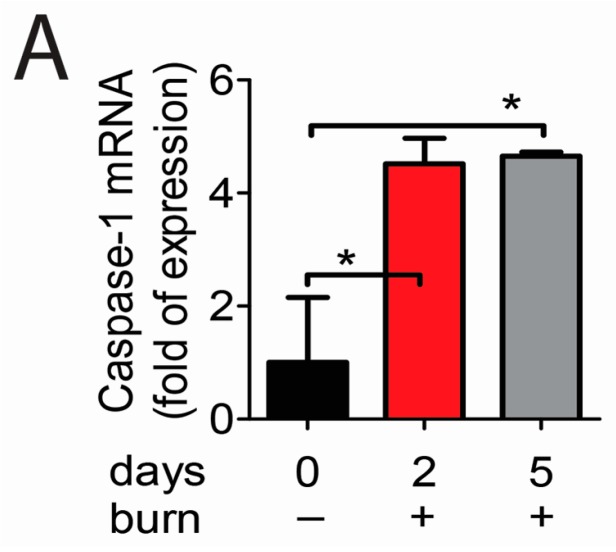

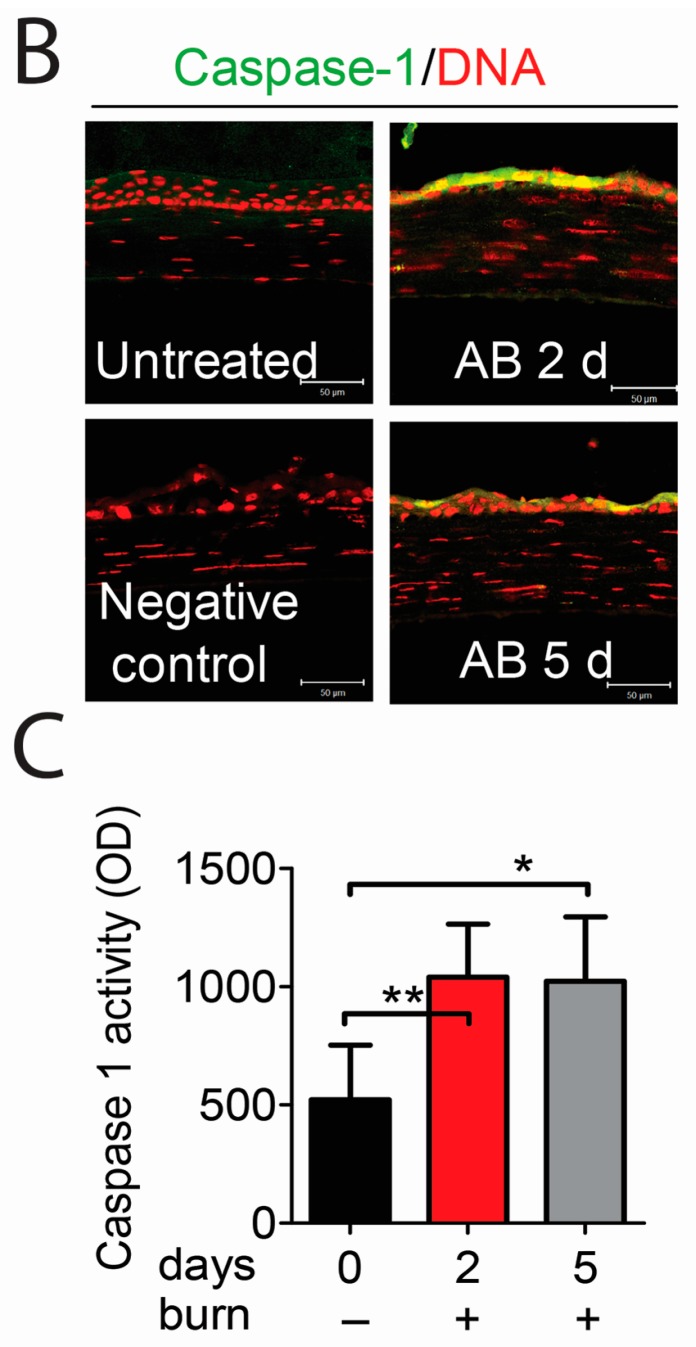
Caspase-1 activation in corneal epithelium in animals subjected to alkali burn (AB) at 2 days (2 d) and 5 days (5 d) post-injury. (**A**) Gene expression analysis of caspase-1 mRNA transcript in whole cornea of animals subjected to alkali burn (*n* = 8 animals/group). Graphs show mean ± standard error of the mean (SEM); * *p* < 0.05; (**B**) Representative merged digital images of laser scanning confocal microscopy in corneal cryosections immunostained for caspase-1 (green) with propidium iodide nuclei counterstaining (DNA in red) in animals subjected to alkali burn (*n* = 6 animals/group). Scale bar: 50 µm; (**C**) Caspase-1 activity in whole cornea lysates from eyes subjected to alkali burn (*n* = 12 animals/3 samples/group). Graphs show mean ± SEM; * *p* < 0.05, ** *p* < 0.01.

Upon interaction of NLRP3 with ASC, the ASC recruits pro-caspase at the caspase activation and recruitment domain (CARD), thus forming active inflammasome complexes in response to a variety of exogenous and endogenous stressors [14,15]. We evaluated the expression of *caspase-1* gene in injured corneas and compared findings with normal corneas. As shown in Figure 3A, alkali-injured corneas had higher expression of caspase-1 transcripts at both 2 and 5 days compared with normal controls.

Immunoreactivity of corneas to caspase-1 was evaluated by immunostaining (Figure 3B). Although barely detected in naïve corneas, increased reactivity against caspase-1 was noted in cornea epithelia of wounded corneas at 2 and 5 days. Caspase-1 activity in the corneal epithelium demonstrated significantly higher levels of activated caspase-1 at both 2 and 5 days compared to untreated corneas (Figure 3C).

These results show that caspase-1 is increased and activated in corneal alkali-burned corneas. Taken together, these findings show that the NLRP3–ASC–caspase-1 inflammasome is assembled in alkali-injured corneas.

### 2.3. The NLRP3–ASC–Caspase-1 Inflammasome Directs IL-1β Secretion in Alkali-Injured Corneas

**Figure 4:**
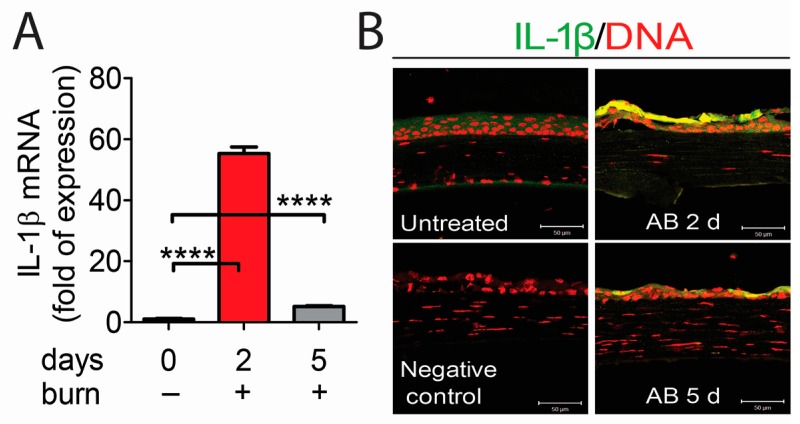
Upregulation of interleukin (IL)-1β in corneal epithelium in animals subjected to alkali burns (AB) at 2 days (2 d) and 5 days (5 d) post-injury. (**A**) Gene expression analysis of IL-1β mRNA transcripts in whole corneas of eyes subjected to alkali burn (*n* = 8 animals/group). **** *p* < 0.0001; (**B**) Representative merged digital images of laser scanning confocal microscopy in corneal cryosections immunostained for IL-1β (green) with propidium iodide nuclei counterstaining (DNA in red) in corneas subjected to alkali burn (*n* = 6 animals/group). Scale bar: 50 µm.

The NLRP3 inflammasome is critical for IL-1β production [16,17]. To investigate whether NLRP3 inflammasome formation can lead to IL-1β production in the alkali corneal burn model, we evaluated the expression of IL-1β in wounded corneas by PCR and immunostaining. As shown in Figure 4A, wounded corneas had higher expression of IL-1β mRNA at 2 and 5 days compared to naïve corneas. Consistent with PCR results, increased reactivity to IL-1β in the corneal epithelium was seen in wounded corneas at 2 and 5 days.

Taken together, our results demonstrated that NLRP3 inflammasome is involved in corneal alkali injury, leading to IL-1β production, which may be critical in inducing an inflammatory response.

### 2.4. Sodium Butyrate and β-Hydroxybutyric Acid Topical Treatment Inhibits Activation of NLRP3 Inflammasome in Alkali-Injured Corneas

Recent studies described NaB and HBA as inhibitors of NLRP3 inflammasome that can inhibit IL-1β release and reduce inflammation [12,18,19]. To test the hypothesis that blocking NLRP3 would improve clinical parameters and reduce inflammation, alkali-burned corneas were treated with either NaB, HBA, or vehicle for 2 or 5 days post-injury. To compare the relative potency of these molecules, a group of alkali-injured corneas treated with dexamethasone (Dex) was also included, as our previous study showed that Dex is very efficacious in preserving corneal clarity and suppressing inflammation in the most severe model of alkali burn and dry eye [20].

**Figure 5:**
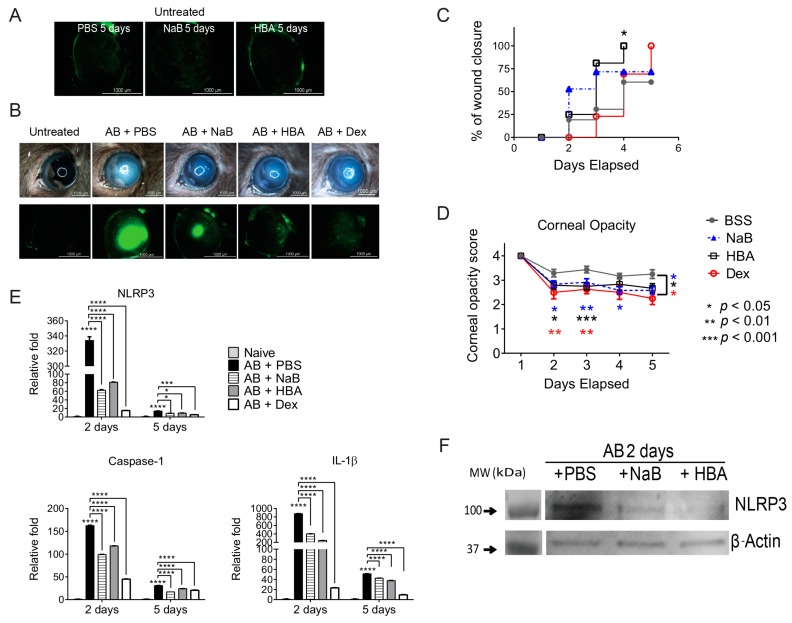
Sodium butyrate (NaB) and hydroxybutyric acid (HBA) eyedrops improve clinical parameters and decrease inflammation in animals subjected to alkali burns (AB). (**A**) Digital images of corneas stained with 0.1% sodium fluorescein demonstrating intact corneal epithelium after topical NaB, HBA or balanced salt solution (BSS) treatment for 5 days quater in die (QID) in untreated mice (*n* = 5 animals/group). Scale bar: 1000 µm; (**B**) Representative color digital images used to score corneal opacity (**top row**) and representative fluorescein stained corneas used to create wound closure rate (**bottom row**) at 5 days (*n* = 15 animals/group) after alkali burn. Scale bar: 1000 µm; (**C**) Corneal opacity in corneas subjected to alkali burn and topically treated with either NaB, HBA, or BSS and compared with dexamethasone (Dex) (*n* = 15 animals/group); (**D**) Wound closure rate in corneas subjected to alkali burn and topically treated with either NaB, HBA, or BSS and compared with Dex (*n* = 15 animals/group); (**E**) Mean ± SEM of results of gene expression analysis of *NLRP3*, *Caspase-1*, and *IL-1β* in whole cornea of animals subjected to alkali burn for 2 or 5 days and topically treated with either NaB, HBA, or BSS and compared with Dex (*n* = 5 animals/group). * *p* < 0.05, *** *p* < 0.001, **** *p* < 0.0001. (**F**) Representative digital images of western blot of NLRP3 and β-actin in cornea epithelium of animals subjected to alkali burn for 2 days and topically treated with either NaB, HBA, or BSS (*n* = 12 animals/3 samples/group).

Since there is no evidence of the safety of NaB and HBA application on the ocular surface, NaB and HBA toxicity to the corneal epithelium was first investigated by topically applying NaB or HBA eyedrops quater in die (QID) in naïve mice. Corneal epithelial integrity was assessed using corneal fluorescein staining after 5 days. As shown in Figure 5A, naïve corneas that received NaB or HBA for 5 days had no corneal epithelial defect, indicating that both HBA and NaB were safe to use in vivo.

Next, mice were subjected to alkali burn (AB) and topically treated with NaB, HBA, or BSS (QID), and clinical parameters of cornea opacity and wound healing were evaluated on a daily basis. Representative color digital images used to score corneal opacity (top row) and fluorescein stained corneas used to measure wound closure rate (bottom row) are shown in Figure 5B. At 5 days post-injury, HBA treated corneas had significantly higher wound closure rate (Figure 5C) and both NaB and HBA treated corneas had lower corneal opacity scores (Figure 5D) compared to vehicle controls, respectively. β-hydroxybutyric acid-treated corneas healed significantly faster than vehicle treated corneas, with 100% wound closure at 4 days post-injury.

To determine if blocking NLRP3 inflammasome can suppress inflammation in alkali-injured corneas, the expression of NLRP3, caspase-1, and IL-1β mRNA transcripts in alkali burned corneas treated with NaB or HBA were evaluated using real-time PCR and compared with vehicle-treated corneas. Polymerase chain reaction results showed that both NaB treatment and HBA treatment after alkali burn significantly decreased NLRP3, caspase-1, and IL-1β mRNA transcripts at both 2 and 5 days compared to BSS-treated corneas (Figure 5E). Western blot confirmed the real-time PCR results with decreased protein expression of NLRP3 in corneal epithelia in AB + HBA and AB + NaB groups compared to AB + vehicle group at 2 days (Figure 5F).

Alkali-burned corneas treated with Dex showed significant improvement in corneal opacity (Figure 5A–C), and impressive decreases in inflammatory cytokines (Figure 5E). Although the anti-inflammatory effect of Dex was greater than NaB and HBA, NaB and HBA treatment can decrease corneal opacity scores to the level of Dex treated groups, indicating that NaB and HBA are very efficacious in preserving corneal clarity. These data indicated that blocking the NLRP3 inflammasome pathway using NaB and HBA drops improves clinical parameters and decreases inflammation in animals subjected to alkali burns at 2 and 5 days post-injury.

## 3. Discussion

In the present study, we demonstrated that corneal epithelial cells participated in innate immunity through the NLRP3–ASC–caspase-1–IL-1β pathway in response to sterile corneal injuries. Blocking the NLRP3 pathway with NaB and HBA in the injured corneas can suppress inflammation and preserve cornea clarity post injury.

Damage to the cornea from an ocular chemical burn is severe, leading to overwhelming sterile inflammatory responses, and corneal epithelial defects or sterile corneal ulceration at the acute stage. The role of corneal epithelium in this inflammatory cascade has not been established. Studies have shown that epithelial cells play a vital role in innate immunity by expressing the main families of PPRs, including TLRs and NLRs, in response to various ocular surface stimuli [6,7,8]. In the context of infection, *Pseudomonas aeruginosa* flagellin elicits inflammatory response of corneal epithelium through the TLR5–nuclear factor κ-light-chain-enhancer of activated B cells (NF-κB) signaling pathway [7]. Human corneal epithelial cells exposed to *Aspergillus fumigatus* can trigger innate immune response via activating NOD 1 receptors on human corneal epithelial cells, leading to the secretion of IL-6, IL-8, and tumor necrosis factor-α (TNF-α). NOD1 knockdown attenuated *Aspergillus fumigatus* triggered expression of *IL-6*, *IL-8*, and *TNF-α* [4].

It is now evident that PPRs can recognize endogenous molecules that are released during cellular injury termed DAMPs. Damage-associated molecular patterns released by dying cells can stimulate severe inflammation [21]. How corneal epithelial cells response to DAMPs and how this process affects the immune network has not been fully elucidated. The NLRP3 inflammasome has been described as an innate sensor of host-derived DAMPs that are released following tissue injury or cell death to activate pro-inflammatory pathways [9]. NOD-like receptor family pyrin domain containing 3 is expressed by hematopoietic and non-hematopoietic cells, such as keratinocyte and osteoblasts. In this study, we showed that the naïve corneal epithelial cells express low levels of NLRP3, while wounded corneal epithelium upregulated NLRP3 expression, indicating that NLRP3 participates in the wound healing process of a sterile corneal injury in response to DAMPs.

NOD-like receptor family pyrin domain containing 3 contains domains that can interact with adaptor protein ASC which has a CARD to recruit pro-caspase-1, thus forming the inflammasome [22,23,24]. Once activated, the NLRP3 inflammasome causes the activation of caspase-1 by cleaving pro-IL-1β into biologically active IL-1β. The adaptor protein ASC and pro-caspase-1 are also crucial to induce caspase-1 activation. Shrikant et al. [25] showed that calcium oxalate crystals induce renal inflammation by NLRP3 mediated IL-1β secretion. Interleukin-1β secretion was found to be reduced in NLRP3^−/−^, ASC^−/−^, and Caspase-1^−/−^ mice. In our study, low levels of ASC and caspase-1 were found in naïve mouse corneas, while there was remarkably increased expression of ASC and caspase-1 in the wounded corneal epithelium, suggesting the activation of NLRP3 inflammasome in the corneal epithelia in response to corneal alkali injuries.

NOD-like receptor family pyrin domain containing 3 inflammasome has been implicated in the pathogenesis of many eye diseases. Zheng and colleagues [26] showed that reactive oxygen species induced NLRP3 expression by the corneal epithelium in a dry eye mouse model. We have also reported that NLRP3 and NLRC4 inflammasomes were activated in cultured human corneal epithelial in response to hyperosmolar stress [27]. In the context of infection, *Staphylococcus aureus* activates the NLRP3 inflammasome in human and rat conjunctival goblet cells [28]. In patients with age-related macular degeneration (AMD), increased mRNA levels of NLRP3, IL-1β, and pro-IL-18 in lesions of the retinal pigment epithelium and photoreceptors were detected [29]. Activation of NLRP3 inflammasome also contributed to retinal ganglion cell death following partial optic nerve crush injury [24] in a mouse model, while NLRP3 deficient mice have significantly reduced neuroinflammation and delayed retinal ganglion cell loss. Therefore, inhibiting NLRP3 assembly would be a novel therapeutic approach to limit many diseases, including eye diseases.

Recently, studies of targeted anti-NLRP3 therapy have opened a new chapter in the treatment of inflammatory disease. The ketone body HBA is produced by hepatocytes and astrocytes as an alternative energy source during fasting or exercise. However, in vivo and in vitro studies have demonstrated that HBA is more than just a metabolite. It has important cellular signaling roles as well. Youm et al. [19] reported HBA specifically inhibits NLRP3 inflammasome activation and decreases the production of active IL-1β and IL-18. Sodium butyrate has a similar chemical structure to HBA and also has been reported to inhibit the NLRP3 pathway [12]. To investigate if blocking NLRP3 pathway activation in injured corneas can improve clinical parameters and suppress inflammation, we used NaB and HBA topically in the alkali-burned eyes and evaluated corneal wound healing and corneal clarity, as well as the expression of NLRP3, caspase-1, and IL-1β in the burned corneas, and compared results with vehicle and Dex treated corneas. Dexamethasone therapy has been proven to be very efficacious in preserving corneal clarity and suppressing inflammation [20]. In this study, we observed that HBA and NaB treatment can preserve cornea clarity at a similar level to Dex treated corneas, indicating that HBA and NaB are efficacious in preserving corneal clarity. In agreement with a study by Nakamura et al. [13] who showed that HBA has a protective effect on corneal epithelia in dry eye conditions, our results showed HBA can promote wound healing at 5 days post-injury. In addition to the improvement of clinical parameters, the expression of the key component of NLRP3 pathway also decreased in HBA and NaB treated corneas. Thus, our results suggest that blocking the NLRP3 pathway leads to improved clinical parameters and suppresses inflammation.

In conclusion, this study reveals that the NLRP3 expressed by corneal epithelial cells may respond to DAMPs released after sterile corneal injury, leading to the production of IL-1β via the innate immune signaling pathway (NLRP3–ASC–caspase-1–IL-1β). We have shown for the first time that NLRP3 inflammasome is involved in the sterile corneal injury and provided mechanistic and therapeutic considerations. Inhibiting NLRP3 assembly would be a novel therapeutic approach to limit cornea damage after alkali burns.

## 4. Materials and Methods

### 4.1. Animals

All animals were treated in accordance with the Association of Research in Vision and Ophthalmology (ARVO) Statement for the Use of Animals in Ophthalmic and Vision Research, and the protocols were approved by the Baylor College of Medicine Institutional Animal Care and Use Committee (IACUC protocol number AN-5076, first approved on 24 October 2011). Female C57BL/6J mice (6–8 weeks old) were purchased from the Jackson Laboratory (Bar Harbor, ME, USA).

### 4.2. Unilateral Alkali Burn

After systemic anesthesia with isoflurane using a vaporizer (SomnoSuite; Kent Scientific, Torrington, CT, USA), a unilateral alkali burn was created on the right eye of 6- to 8-week-old C57BL/6 mice. This was achieved by placing one 2-mm diameter filter paper disc that had been presoaked with 1 N NaOH on the central cornea for 10 s, followed by extensive rinsing with balanced salt solution (Alcon, Fort Worth, TX, USA), as previously described [20]. Precautions were taken to avoid damage to the peripheral cornea, conjunctiva, and lids. Alkali burn was created at day 0 and animals were euthanized 2 or 5 days post-injury.

### 4.3. Histology and Immunostaining

For immunohistochemistry, eyes and adnexae from each group/time point (*n* = 6/group) were excised, embedded in optimal cutting temperature compound (VWR, Suwanee, GA, USA), and flash frozen in liquid nitrogen. Sagittal 8 µm tissue sections were cut with a cryostat (HM 500; Micron, Waldorf, Germany) and placed on glass slides that were stored at −80 °C.

Immunofluorescent staining was performed in frozen tissue sections with rat monoclonal antibody anti-NLRP3 (MAB7578, 10 µg/mL, R&D Systems, Minneapolis, MN, USA), anti-ASC (SC-22514-R, 1 µg/mL, Santa Cruz Biotechnology, Dallas, TX, USA), anti-caspase-1 (SC-56036, 1 µg/mL, Santa Cruz Biotechnology, Dallas, TX, USA), and goat anti-IL-1β (1:50 dilution, #12426, Cell Signaling Technology, Beverly, MA, USA). Secondary goat anti-rabbit or donkey anti-goat Alexa Fluor 488-conjugated antibodies were used, as previously described [30]. The images were captured and photographed by a laser scanning confocal microscope (LSM 510, with Kr–Ar and He-Ne laser; Carl Zeiss Meditec, Inc., Thornwood, NY, USA).

### 4.4. RNA Isolation and Quantitative PCR

Whole corneas or corneal epithelium (*n* = 4/group per experiment; total of eight corneas/group) were collected and minced, and total RNA was extracted using a Qiagen MicroPlus RNeasy isolation Kit (Qiagen, Valencia, CA, USA) according to the manufacturer’s instructions, quantified by a NanoDrop ND-2000 Spectrophotometer (Thermo Fisher Scientific, Wilmington, DE, USA), and stored at −80 °C. First-strand complement DNA was synthesized with random hexamers by M-MuLV reverse transcription (Ready-To-Go You-Prime First-Strand Beads; GE Healthcare, Inc., Arlington Heights, NJ, USA), as previously described [20].

Real-time PCR was performed with specific Taqman MGB probes (Applied Biosystems, Inc., Foster City, CA, USA) and PCR master mix (Taqman Gene Expression Master Mix), in a commercial thermocycling system (StepOnePlus Real-Time PCR System, Applied Biosystems, Inc.), according to the manufacturer’s recommendations. Quantitative real time PCR was performed using gene expression assay primers and probes specific for murine targets, as described in Table 1. The β-2-microglobulin (B2M) gene was used as an endogenous reference for each reaction to correct for differences in the amount of total RNA added. The results of quantitative PCR were analyzed by the comparative cycle threshold (CT) method where the target change equals 2^–ΔΔCT^. The results were normalized by the CT value of B2M and the relative mRNA level in the untreated group was used as the calibrator.

### 4.5. Caspase-1 Activation Fluorometric Assays

The activation of caspase-1 was measured in corneal epithelial lysates according to the manufacturer’s protocol (K110-200, BioVision, Inc., Mountain View, CA, USA). One sample was pooled from four right corneas and there were a total of three samples per time point. Total protein concentration was measured by the bicinchoninic acid (BCA) protein assay (Thermo Fisher Scientific, Waltham, MA, USA), as previous described [32]. Three samples per group were used. Caspase-1 activities were measured (50 µg/sample) by following the cleavage of the fluorescent substrate analogs in a fluorescent plate reader (Tecan Infinite M200, Magellan V6.55 software, Tecan, Männedorf, Switzerland) with 400 nm excitation filter and 505 nm emission filter. The results were exported and averaged.

### 4.6. Western Blot

Corneal epithelium was scraped with a scalpel and placed in 100 μL RIPA buffer (R0278; Sigma–Aldrich, St. Louis, MO, USA) with protease inhibitor cocktail (11836170001, Roche, Basel, Switzerland). One sample was pooled from four right corneas and there were a total of three samples per time point. A BCA assay was performed to measure total protein concentration of each sample. Samples (25 μL, equal to a protein concentration 50 μg) were diluted with one part 2X sample buffer (2X Laemmli Sample Buffer, 161-0737; Bio-Rad Laboratories, Inc., Hercules, CA, USA), boiled for 5 min, and loaded on to the polyacrylamide gel. The gel (mini-PROTEAN TGX stain free Precast Gel 7.5%, 456-8024; Bio-Rad laboratories, Inc., Hercules, CA, USA) was run at constant current at 100V for approximately 90 min at room temperature before the proteins were transferred onto a polyvinylidene difluoride (PVDF) membrane (Immobilion Transfer membranes, IVPH07850; Millipore, Billerica, MA, USA) at 20 V over night at 4 °C. Next, the membranes were incubated in 100 mM Tris-HCl, 0.9% NaCl, 0.1% Tween 20 (TTBS) with 5% fat-free milk for 60 min, followed by incubation in primary antibody NLRP3 (MAB7578, 2 µg/mL, R&D Systems, Minneapolis, MN, USA) or β-actin (#4970, 1 µg/mL, Cell Signaling technology, Beverly, MA, USA) overnight at 4 °C. Subsequently, the membranes were washed in TTBS and incubated in secondary horseradish peroxidase goat anti-rat (629520; Thermo Fisher Scientific), then washed again with TTBS, and finally, developed and photographed.

### 4.7. Treatment Regimen

Mice subjected to corneal alkali burn were topically treated either with 2 µL of sodium butyrate (0.5 Mm, Sigma–Aldrich), 2 µL of hydroxybutyric acid (80 mM, Sigma–Aldrich), sodium phosphate dexamethasone (0.1%, Spectrum Laboratory, Gardena, CA, USA), or vehicle (balanced salt solution, Alcon, Fort Worth, TX, USA) QID for 2 or 5 days. These doses were chosen based on published manuscripts [12,13,19,33] and also based on our pilot studies.

### 4.8. Clinical Findings: Opacity Score

Biomicroscopic examination was used to grade corneal edema and opacity by two masked observers in images taken by a color digital camera DS-Fi1 (Melville, NY, USA) by the way described by Yoeruek (2008) [34]. Corneal opacity was scored using a scale of 0–4 where grade 0 represented completely clear conditions; grade 1 slightly hazy, iris, and pupils easily visible; grade 2 slightly opaque, iris, and pupils still detectable; grade 3 opaque, pupils hardly detectable, and grade 4 completely opaque with no view of the pupils.

### 4.9. Measurement of Corneal Epithelial Defect

Corneal epithelial healing was assessed daily in the experimental groups (four mice per group per experiment; three sets of experiments). Briefly, 1 mL of 0.1% liquid sodium fluorescein was instilled onto the ocular surface. Corneas were rinsed with phosphate-buffered saline and photographed with a stereoscopic zoom microscope (SMZ 1500; Nikon, Melville, NY, USA) under fluorescence excitation at 470 nm (digital camera DS-Qi1Mc, Nikon). Corneal epithelial defect area was graded in digital images by two masked observers in a categorical manner (present/absent) to generate a survival curve [35]. Biological replicate scores were transferred to an Excel database (Microsoft, Redmond, WA, USA) and results analyzed.

### 4.10. Number of Animals and Statistical Analysis

One hundred and thirty-three C57BL/6J mice were used in this study. Fifty-eight animals were used per time point (2 and 5 days): 6 for histology, 16 for real-time PCR, 12 for caspase-1 activity assay, and 24 for western blot. Contralateral eyes in the alkali burn group were used as untreated controls. Fifteen naïve mice were used to evaluate drug toxicity.

Results are presented as the mean ± SEM. One-way analysis of variance (ANOVA) with Bonferroni post hoc testing was used for statistical comparisons of gene expression. *p* ≤ 0.05 was considered statistical significant. These tests were performed using GraphPad Prism 6.0 software (GraphPad Incorporation, San Diego, CA, USA).

## Figures and Tables

**Table 1:** Oligonucleotide primers used for real-time PCR.

Gene Name	Symbol	Assay ID *
β-2-microglobulin	*B2M*	Mm00437762
Caspase-1	*Caspase-1*	Mm00438023
Interleukin-1β	*IL-1β*	Mm00434228
NLR family, pyrin domain containing 3	*NLRP3*	Mm00840904

* Identification number from Thermo Fisher Scientific [31].

## Abbreviations

AB	alkali burn
d	days
UT	untreated
IL	interleukin
NaB	Sodium butyrate
HBA	β-hydroxybutyric acid
BSS	balanced salt solution
ASC	apoptosis-associated speck-like protein
B2M	beta-2-microglobulin
BCA	bicinchoninic acid
Dex	dexamethasone
NOD	nucleotide-binding oligomerization domain-containing protein
NLR	NOD-like receptor
PAMPs	pathogen-associated molecular patterns
TLR	Toll like receptor
DAMPs	damage-associated molecular patterns
ANOVA	one-way analysis of variance
CARD	caspase activation and recruitment domain
QID	quater in die (in Latin, four times a day)
